# Franck-Condon Factors to High Vibrational Quantum Numbers I: N_2_ and $N_{2}^{+}$

**DOI:** 10.6028/jres.065A.047

**Published:** 1961-10-01

**Authors:** R. W. Nicholls

## Abstract

Franck-Condon factor arrays have been computed numerically to high vibrational quantum numbers for the band systems

$$\begin{array}{l} N_{2}:\;C^{3}\text{II}-B^{3}\text{II}\;(\text{Second Positive})\\ N_{2}:\;B^{3}\text{II}-A^{3}\sum \;(\text{First Positive})\\ N_{2}:\;A^{3}\sum -X^{1}\sum \;(\text{Vegard Kaplan})\\ N_{2}:\;a^{1}\text{II}-X^{1}\sum \;(\text{Lyman-Birge-Hopfield})\\ N_{2}^{+};\;A^{2}\text{II}-X^{2}\sum \;(\text{Meinel})\\ N_{2}^{+}:\;B^{2}\sum -X^{2}\sum \;(\text{First Negative}) \end{array}$$

and for the following ionization transitions

$$\begin{array}{l} N_{2}\;X^{1}\sum \to N_{2}^{+}X^{2}\sum \\ N_{2}\;X^{1}\sum \to N_{2}^{+}A^{2}\text{II}\\ N_{2}\;X^{1}\sum \to N_{2}^{+}B^{2}\sum \end{array}$$

## 1. Introduction

The intensity $I_{v^{\prime }v^{\prime \prime }}$ in emission of the (*v*′,v″) molecular band is given by Nicholls (1958)

$$I_{v^{\prime }v^{\prime \prime }}=KN_{v\prime }E_{v^{\prime }v^{\prime \prime }}^{4}R_{e}^{2}\left({\bar{r}}_{v^{\prime }v^{\prime \prime }}\right)q_{v^{\prime }v^{\prime \prime }}$$ (1)

where *K* is a constant which contains the effects of geometry and units, *N_v_*_′_ is the population in the level *v′, E_v_*_′_*_v_*_″_, is the energy quantum of the *v′,v″* transition, *R_e_*(*r*) is the electronic transition moment, *r* is the internuclear separation and ${\bar{r}}_{v^{\prime }v^{\prime \prime }}$ is the *r*-centroid (Nicholls and Jarmain, 1956, 1959) of the transition and *q_v_*_′_*_v_*_″_ is the Franck-Condon factor (Bates, 1952; Nicholls, 1958). *R_e_*(*r*), ${\bar{r}}_{v^{\prime }v^{\prime \prime }}$ and *q_v_*_′_*_v_*_″_ are respectively defined by:

$$R_{e}\left(r\right)=\int \psi_{e}^{\prime *}M\psi_{e}^{\prime \prime }d\tau_{e}$$ (2)

$${\bar{r}}_{v^{\prime }v^{\prime \prime }}=\int \psi_{v^{\prime }}\psi_{v^{\prime \prime }}rdr/\int \psi_{v^{\prime }}\psi_{v^{\prime \prime }}dr$$ (3)

$$q_{v^{\prime }v^{\prime \prime }}={\left|\int \psi_{v^{\prime }}\psi_{v^{\prime \prime }}dr\right|}^{2}$$ (4)

where $\psi_{e}^{\prime },\psi_{e}^{\prime \prime }$ are respectively electronic wave functions of the upper and lower states involved, *M* is the multipole moment of the transition, *dτ_e_* is the element of configuration space of electronic wave function, *r* is the internuclear separation, and *ψ_v_*_′_*ψ_v″_* are the vibrational wave functions of the upper (*v*^′^) level and lower (*v″*) level, respectively.

The Franck-Condon factor (*q_v′v_*_″_) is governed by the overlap of the vibrational wave functions of the two levels which are concerned in the *v′,v″* transition. It is sometimes called, somewhat clumsily, the vibrational overlap integral square and can vary over two or three orders of magnitude for the significant bands of a system. For many band systems, although the variation from band to band of $N_{v^{\prime }},E_{v^{\prime }v^{\prime \prime }}^{4}$ and *R_e_*(*r*) is significant, their combined effect is often not more than one or two orders of magnitude. *E* does not change very much over a band system of relatively small wavelength extension (e.g., Δ*E*^4^/*E*^4^= 14 percent between 2000 and 6000A and few band systems are so extensive). *N_v_*_′_ is of course controlled by excitation conditions. For a number of band systems which have been studied, a significant variation *R_e_*(*r*) with *r* has been measured, (Turner and Nicholls, 1954a, b; Wallace and Nicholls, 1955; Nicholls 1956; Dixon and Nicholls, 1958; Robinson and Nicholls, 1958, 1960; Hebert and Nicholls, 1961). It is however seldom as much as an order of magnitude. Thus the Franck-Condon factor exerts dominant effect upon the intensity variation from hand to band across a system.

Since the pioneering work of Hutchisson (1930, 1931) who used an analytic method to evaluate the vibrational overlap integral for homonuclear molecules (harmonic and anharmonic oscillators), and its extension by Dunham (1932) to heteronuclear molecules, a number of authors have used a wide variety of methods and have produced a number of *q_v′v_*_″_ arrays of varying extent. This work may be summarized as follows:

Gaydon and Pearse (1939) proposed a method by which wave functions of a simple harmonic oscillator are linearly distorted to fit an equivalent Morse Potential. They compared Franck-Condon factors calculated in this way with their experimental results on RbH. Pillow (1949, 1950, 1951, 1952, 1953a, b, 1954, 1955) has improved and applied the method to a large number of transitions (Pillow and Rowlatt, 1960). Nicholls (1950), Montgomery and Nicholls (1951) and Turner and Nicholls (1951) also used the method.

The basis of the distortion method was criticized by Wu (1952) who suggested two further analytic methods for Morse molecules. One of these methods which is based on the WKB approximation has been used by Wyller (1953, 1958). Bates (1952) has published a very useful double entry table based upon modifications of Hutchisson’s formulas, from which Franck-Condon factors for any transition may be read *v′*=0*—*2: *v″* = 0—2.

Prior to the general availability of digital computers, the extreme tedium of direct numerical integration, such as has been carried out by Bates (1949) and Jarmain and Nicholls (1954) for Morse molecules, forced most workers to resort to analytic approximations to overlap integrals. Most of these approximations are valid for small quantum numbers. An example is the analytic method of Fraser and Jarmain (Fraser and Jarmain, 1953; Jarmain and Fraser, 1953; Fraser, 1954) which has been applied to a wide variety of band systems (Jarmain, Fraser, and Nicholls, 1953, 1955; Fraser, Jarmain, and Nicholls, 1954; Nicholls, Fraser, and Jarmain, 1959; Nicholls, Fraser, and Jarmain, and McEachran, 1960; Jarmain, Ebisuzaki, and Nicholls, 1960; Ortenberg, 1960).

Biberman and Yakubov (1960) have recently proposed a WKB method of evaluating Franck-Condon factors analytically at high vibrational quantum numbers. The method has been applied by Yakubov (1960). Nevertheless, with the current availability of large capacity electronic computers, direct numerical integration to high quantum numbers is straightforward, and there seems to be little point in developing further approximate analytic methods. The only limitation of this method is the realism (particularly at high quantum numbers) of the empirical potentials used.

Losev (1958) used a small capacity computer for direct evaluation of overlap integrals. The capacity of the machine was not, however, sufficient to accommodate the severe cancellation.

Ideally, Franck-Condon factor arrays computed to as high vibrational quantum numbers *v′* and *v″* as are physically reasonable from observed spectra are required for all important molecular band systems. This implies good knowledge of molecular potentials so that the wave functions used are appropriate to ‘real’ rather than empirical potentials. Most of the Franck-Condon factor tables currently in the literature have been calculated for empirical (e.g., simple harmonic or Morse oscillator) potentials.

However recent work of Jarmain (1959, 1960) and Vanderslice et al. (Vanderslice, Mason and Maisch, 1959, 1960a, 1960b; Tobias, Fallon, and Vanderslice, 1960; Fallon, Tobias, and Vanderslice, 1961) has provided, by a WKB-Klein-Dunham method, numerical information on ‘real’ molecular potentials. It is therefore planned to program a numerical solution of the Schrodinger equation appropriate to such numerical potentials to provide numerical wave functions from which more realistic Franck-Condon factor arrays can be determined.

## 2. Method

In the meantime the results reported in this paper represent an interim step. Morse potentials have been assumed and a program for the numerical integration of the overlap integrals has been written for the electronic digital 704 computer at the National Bureau of Standards. The input data are *ω_e_*, *ω_e_x_e_*, *r_e_, μ_A_, v_max_* for both of the electronic states involved. Each member of the two families of wave functions $\psi_{i}^{\prime }(i=0-v_{max}^{\prime })$, $\psi_{j}^{\prime \prime }(j=0-v_{max}^{\prime \prime })$ is computed at 0.01A intervals, overlap integrals between all pairs of them are computed and squared. The program has been applied to a number of transitions and results are presented here for transitions in *N*_2_ and $N_{2}^{+}$.

The basis of the method is as follows: The zeroth vibrational wave function of a Morse molecule is (Morse 1929).

$$\psi_{0}(r)={\left(\frac{\alpha }{\Gamma (K-1)}\right)}^{1/2}{\left[K\exp\;(-\alpha \;{\bar{r-r}}_{e})\right]}^{1/2(K-1)}\exp\;\left[-\frac{K}{2}\exp(-\alpha \;{\bar{r-r}}_{e})\right]$$ (5)

where $K=\frac{\omega_{e}}{\omega_{e}x_{e}}$

$$\alpha =0.243534{\left(\mu_{A}\omega_{e}x_{e}\right)}^{1/2}\;A^{-1}$$

*r_e_*= equilibrium internuclear separation (*A*)

Thus the input data for point by point computation of *ψ*_0_(r) at a series of values of *r* is *ω_e_*, *ω_e_x_e_, r_e_*, *μ_A_.* The symbolism is standard (Herzberg, 1950). The gamma function may be evaluated through Stirling’s formula:

$$\Gamma (x)={\left(\frac{2\pi }{x}\right)}^{1/2}x^{x}\exp(-x)\left\{1+\frac{1}{12x}+\frac{1}{288x^{2}}+\cdots \right\}$$ (6)

Once the zeroth wave function is tabulated the complete family of wave functions can be built up one at a time by the relationships

$$\left(\frac{\psi_{v}}{\psi_{v-1}}\right)=\left(\frac{C_{v}}{C_{v-1}}\right)\frac{1}{Z}\left(\frac{L_{v}}{L_{v-1}}\right)$$ (7)

$$={\left[\frac{\left(K-v)(K-2v-1\right)}{v(K-2v+1)}\right]}^{1/2}\times \frac{1}{K\exp(-\alpha \;{\bar{r-r}}_{e})}\left(\frac{L_{v}}{L_{v-1}}\right)$$ (8)

$$C_{0}=\left(\frac{\alpha }{\Gamma (K-1)}\right)\cdot^{1/2}$$ (9)

The wave functions *ψ_v_* of higher quantum number than zero contain, as is seen in eq (7), ratios of associated La guerre functions *L_v_* of the form:

$$L_{v}=L_{K-v-1}^{K-2v-1}(Z)=Z^{v}-v(K-v-1)Z^{v-1}+\frac{v(v-1)}{2}(K-v-1)(K-v-2)Z^{v-2}+\ldots +{\left(-1\right)}^{v}\{(K-v-1)(K-v-2)\ldots (K-2v)\}$$ (10)

where the number of terms in the *v*th polynomial is (*v*+1); $L_{0}=L_{K-1}^{K-1}\left(Z\right)=1]$; $Z=K\;\exp\left(-\alpha \;{\bar{r-r}}_{e}\right)$. For the lowest quantum numbers, eq (10) may be used in the evaluation of *L_v_.* At higher values of *v* loss of significant figures through cancellation between positive and negative terms forces the use of the recursion relation

$$L_{v}\left(Z\right)=ZL_{v-1}-(K-2v)\sum_{r=0}^{v-1}\left(\begin{array}{c} v-1\\ r \end{array}\right)\frac{(2r)!}{(r+1)!}L_{v-r-1}$$ (11)

(Fraser, Jarmain, and Henderson, 1959). If in the use of eq (8)*L_v_*_−1_ should vanish at some value of *r*, the alternative relation

$$\left(\frac{\psi_{v}}{\psi_{v-2}}\right)=\left(\frac{C_{v}}{C_{v-2}}\right)\left(\frac{1}{Z^{2}}\right)\left(\frac{L_{v}}{L_{v-2}}\right)$$ (12)

can be used.

Prior to numerical integration, each wave function is evaluated at 0.01A intervals over its significant range of *r.* This significant range may be estimated by recalling that the width *r*_2_*—r*_1_ of a Morse potential in the region of energy *U* is given by

$$\;r_{2}-r_{1}=\frac{1}{\alpha }\log_{e}\left[\frac{1+{\left(\frac{U}{D}\right)}^{1/2}}{1-{\left(\frac{U}{D}\right)}^{1/2}}\right]=\frac{1}{\alpha }\log_{e}\left(\frac{1+2{\left(V-V^{2}\right)}^{1/2}}{1-2{\left(V-V^{2}\right)}^{1/2}}\right)\text{at level}\;v\;\text{where}\;V=x_{e}(v+1/2).$$ (13)

Rough rules of thumb for the total range in *r* to be treated are for many electronic states

*v*_max_~6 Add 50% to *r*_2_*−r*_1_: 40% of total range should be less than *r_e_*
*v*_max_~12 Add 40% to *r*_2_*−r*_1_*:* 40% of range should be less than *r_e_*
*v*_max_~18 Add 30% to *r*_2_*−r*_1_*:* 35% of range should be less than *r_e_.*

Beyond these ranges the amplitudes of wave functions are negligible.

Provision has been made for storage on magnetic tape of the vibrational wave functions involved in the NBS computer center, for future users. Checks were of course applied for normality of the wave functions i.e., ∫(*ψ_v_*)^2^ *dr*=1 and the overlap integrals between all possible pairs of wave functions of a transition were evaluated. The overlap integrals were squared and values checked by applying the sum rules Σ*_v′_q_v′v″_* = 1 = Σ*_v″_q_v′v″_*. It may be noted in passing that computation of *r*-centroids was also made. These will be discussed independently elsewhere.

## 3. Basic Data and Results

Franck-Condon factor arrays have been computed for the following radiative transitions

$$\begin{array}{l} N_{2}:\;C^{3}{\text{II}}_{u}-B^{3}{\text{II}}_{g}\;\text{Second Positive}\\ \;B^{3}{\text{II}}_{g}-A^{3}\sum_{u}^{+}\;\text{First Positive}\\ \;A^{3}\sum_{u}^{+}-X^{1}\sum_{g}^{+}\;\text{Vegard-Kaplan}\\ \;a^{1}{\text{II}}_{g}-X^{1}\sum_{g}^{+}\;\text{Lyman-Birge-Hopfield}\\ N_{2}^{+}:\;A^{2}{\text{II}}_{u}-X^{2}\sum_{g}^{+}\;\text{Meinel}\\ \;B^{2}\sum_{u}^{+}-X^{2}\sum_{g}^{+}\;\text{First Negative} \end{array}$$

and also for the following ionization transitions

$$\begin{array}{l} N_{2}(X^{1}\sum_{g}^{+},v=0)\to N_{2}^{+}X^{2}\sum (v=0-21)\\ N_{2}(X^{1}\sum_{g}^{+},v=0)\to N_{2}^{+}A^{2}\text{II}(v=0-5)\\ N_{2}(X^{1}\sum_{g}^{+},v=0)\to N_{2}^{+}B^{2}\sum (v=0-29) \end{array}$$

Wave functions were computed in all cases on the basis of a Morse model for as many levels as were known spectroscopically and a rectangular *q_v′v″_* array of (*v*′*_max_*+1) (*v″_max_*+1) members were then evaluated for each transition. In such arrays data was of course calculated for many bands which were not usually spectroscopically observed.

The input data (*ω_e_*, *ω_e_x_e_, r_e_, μ_A_*, *v_max_*) used for each of the states is listed in table 1 and was taken in large part from the compilation of Mulliken (1959) and the original papers of analysis of the band systems.

The nine arrays of Franck-Condon factors are presented in tables 2 to 10 inclusive. [Table t3-jresv65an5p451_a1b] [Table t4-jresv65an5p451_a1b] [Table t5-jresv65an5p451_a1b] [Table t6-jresv65an5p451_a1b] [Table t7-jresv65an5p451_a1b] [Table t8-jresv65an5p451_a1b] [Table t9-jresv65an5p451_a1b]

## 4. Discussion

The most obvious feature of the double entry tables of Franck-Condon factors is, as indicated on them, the family of well defined loci on the *v′ v″* plane of maximum values of the factors. The loci, which have the general appearance of co-axial parabolas correlate well with the occurrence of observed bands. These primary and subsidiary ‘Condon Parabolas’, as the loci are called, pass through those *v′, v″* for which the largest contribution to each overlap integral arises from the strong overlap in *r* between *one* antinode *of each* of the wave functions *ψ_v′_* and *ψ_v″_.*

The wave function *ψ_v_*(*r*) has *v*+1 antinodes of which the general one is $r_{i}^{\left(v\right)}$. $r_{1}^{\left(v\right)}$ and $r_{v+1}^{\left(v\right)}$ are the terminal antinodes and $r_{1}^{\left(v\right)}<r_{e}<r_{v+1}^{\left(v\right)}$. The primary Condon parabolas arise, as is well known, from overlap of the terminal antinodes of *ψ_v′_* and *ψ_v″_.* The two branches of the parabola are determined by the conditions

$$\begin{array}{c} r_{v^{\prime }+1}^{\left(v^{\prime }\right)}=r_{v^{\prime \prime }+1}^{\left(v^{\prime \prime }\right)}\\ r_{1}^{\left(v^{\prime }\right)}=r_{1}^{\left(v^{\prime \prime }\right)} \end{array}\}$$ (14)

The secondary parabolas are determined by overlap of the terminal antinode of one wave function and the next-to-terminal antinode of the other, that is:

$$\begin{array}{c} r_{v^{\prime }}^{\left(v^{\prime }\right)}=r_{v^{\prime \prime }+1}^{\left(v^{\prime \prime }\right)}\\ r_{1}^{\left(v^{\prime }\right)}=r_{2}^{\left(v^{\prime \prime }\right)}\cdot \end{array}\}$$ (15)

The conditions for the *i*th subsidiary parabola are then:

$$\begin{array}{c} r_{v^{\prime }+2-i}^{\left(v^{\prime }\right)}=r_{v^{\prime \prime }+1}^{\left(v^{\prime \prime }\right)}\\ r_{1}^{\left(v^{\prime }\right)}=r_{i}^{\left(v^{\prime \prime }\right)}\cdot \end{array}\}$$ (16)

The geometry of the parabolas has been studied for a variety of potentials by applying the conditions of eqs (14), (15), and (16) and is discussed in detail elsewhere (Nicholls, 1961). In this work the dominating influence of Δ*r_e_* upon the shape of the ‘Parabolas’ is shown analytically. The loci are only true parabolas for simple harmonic potentials as Condon (1926) pointed out for the primary parabola. For Morse Potentials the primary ‘parabola’ is actually a quartic.

The following qualitative statements may be made upon the dominant influence of Δ*r_e_* upon the shape of the loci in particular and upon the form of the three dimensional Franck-Condon factor surface in general.

For band systems in which Δ*r_e_* is quite small, that is, the potentials lie essentially one above the other, the primary parabola is very narrow, appears to lie along the prime diagonal *v′=v″* in the *v′, v″* plane and has its maximum value at the (0,0) band. In a three dimensional representation when Δ*r_e_* is small (~0.01A) the *q_v′v″_* surface is often a descending diagonal ridge along *v′=v″.*

When Δ*r_e_* is a little larger (~0.05A) the ridge widens into a very narrow parabolic ridge with an axial valley. This situation occurs for the $N_{2}^{+}$ First Negative band system.

As Δ*r_e_* is further increased, the primary parabola widens, its vertex moves down the prime diagonal away from (0,0), and subsidiary parabolas appear. At relatively large (~0.4A) values of Δ*r_e_* (e.g., 0_2_ Schumann-Runge and *N*_2_ Vegard-Kaplan systems) the band system is quite extended in wavelength and the oscillator strength of the whole system is spread over many more bands than for systems having a smaller Δ*r_e_.* Thus the intensity per band is decreased.

In an extreme case, the primary parabola lies along the *v′*=0 and *v″*=0 progressions at large *v″* and *v′* respectively and avoids the (0,0) region entirely.

Examples of these general remarks are seen in the tables. The $N_{2}^{+}$ First Negative system having a small Δ*r_e_* (0.043A) exhibits a very narrow primary parabola. The $N_{2}^{+}$ Meinel and *N*_2_ Second Positive systems have nearly equal Δ*r_e_* values (0.061A, 0.064A, respectively) and exhibit somewhat wider primary parabolas and the start of a secondary parabola. The *N*_2_ First Positive system exhibits one primary and two subsidiary parabolas all of which are relatively narrow as Δ*r*_e_=0.08lA for this system. Four wider subsidiary parabolas and one wider primary parabola whose vertex just avoids the (0,0) band are possessed by the *N*_2_ Lyman-Birge-Hopfield system (Δ*r*_e_=0.13A). The extreme pattern of behavior (for Δ*r*_e_=0.196A) is shown by the *N*_2_ Vegard-Kaplan system which possesses six very wide subsidiary parabolas and one wide primary parabola.

A steady diminution in Franck-Condon factors with increasing *v′* is seen in the tables for the *N*_2_ *X*^1^Σ, $N_{2}^{+}$ *X*^2^Σ, $N_{2}^{+}$ *A*^2^II, $N_{2}^{+}$ *B*^2^Σ ionization excitations.

## Figures and Tables

**Table 1:** Basic Data

State	*ω*_e_(cm^−1^)×10^−3^	*ω_e_x_e_*(cm^−1^)×10^−1^	*r_e_*(A)	*μ_A_*	*v_max_*
*N*_2_X^1^Σ	2.35807	1.419	1.0976	7.00377	27
*a*^1^II	1.6937	1.383	1.220	7.00377	16
*A*^3^Σ	1.46037	1.3891	1.293	7.00377	16
*B*^3^II	1.73411	1.447	1.2123	7.00377	21
*C*^3^*II*	2.0351	1.708	1.1482	7.00377	4
$N_{2}^{+}X^{2}\sum$	2.20719	1.614	1.118	7.00363	21
*A*^2^II	1.90284	1.491	1.177	7.00363	5
*A*^2^Σ	2.41984	2.319	1.075	7.00363	29

**Table 2:** Franck-Condon factors to high vibrational quantum numbers for the N_2_ second positive (*C*^3^*II*–*B*^3^*II*) band system

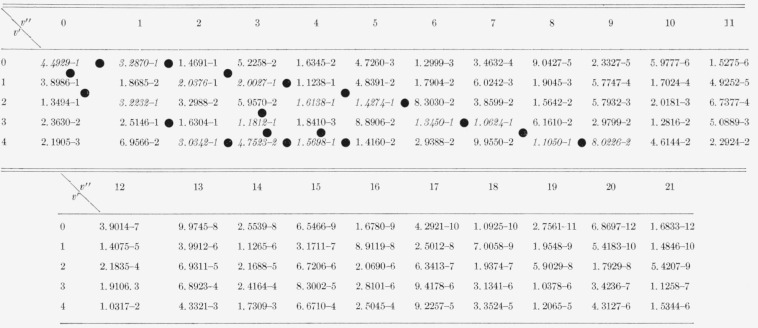

**Table 3 t3-jresv65an5p451_a1b:** Franck-Condon factors to high vibrational quantum numbers for the N_2_ first positive (B^3^*Π*−A^3^Σ) band system

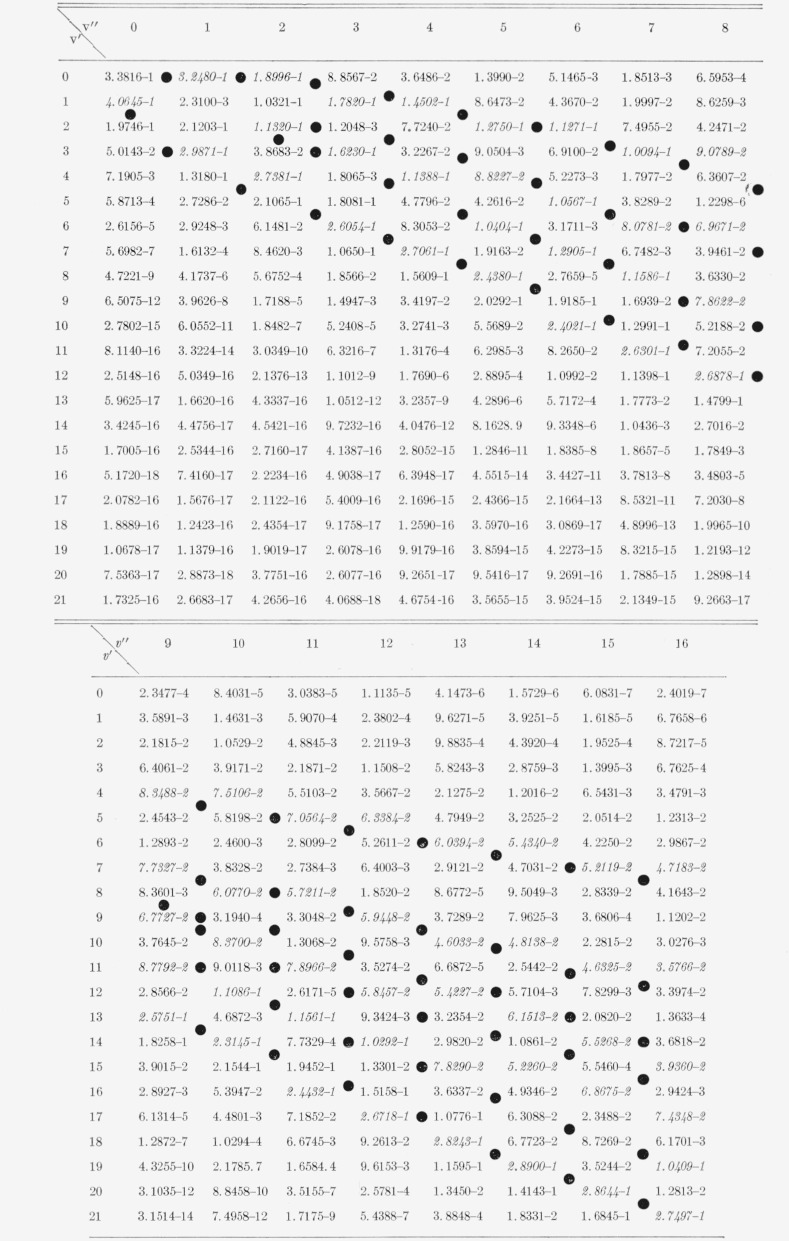

**Table 4 t4-jresv65an5p451_a1b:** Franck-Condon factors to high vibrational quantum numbers for the *N*_2_ Vegard-Kaplan $\left(A^{3}\sum_{g}^{+}-X^{1}\sum_{g}^{+}\right)$ band system

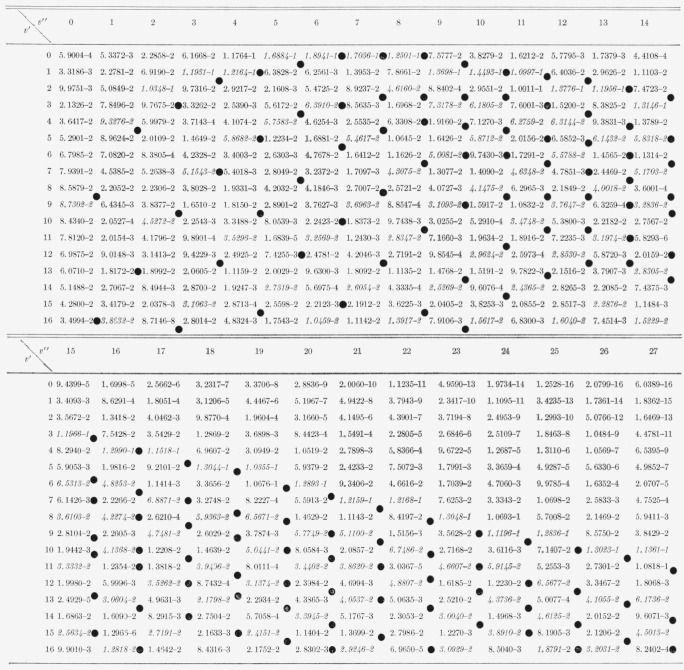

**Table 5 t5-jresv65an5p451_a1b:** Franck-Condon factors to high vibrational quantum numbers for bands of the N_2_ Lyman-Birge-Hopfield $\left(a^{1}{\text{II}}_{g}-X^{1}\sum_{g}^{+}\right)$ system

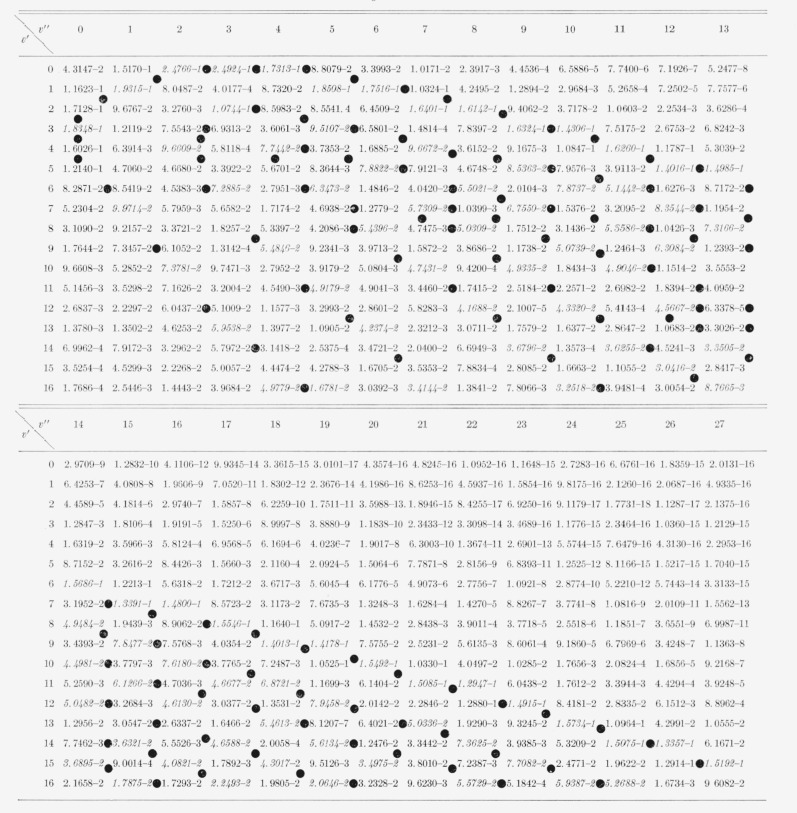

**Table 6 t6-jresv65an5p451_a1b:** Franck-Condon factors to high vibrational quantum numbers for the $N_{2}^{+}$ Meinel (*A^2^II−X^2^Σ*) band system

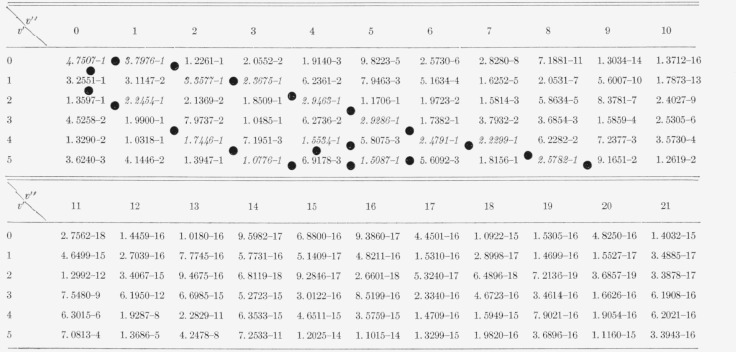

**Table 7 t7-jresv65an5p451_a1b:** Franck-Condon factor array to large vibrational quantum numbers for the $N_{2}^{+}$ first negative (B^2^*Σ*−X^2^*Σ*) band system

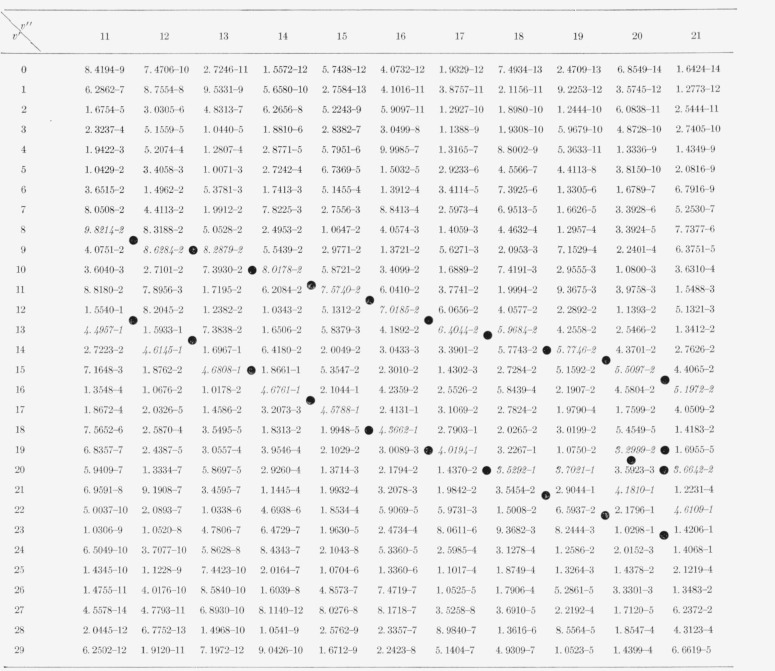

**Table 8 t8-jresv65an5p451_a1b:** Franck-Condon Factors for excitation of levels of N_2_^+^ (X^2^*Σ_g_^+^*) from N_2_(X^1^*Σ_g_^+^*, v = 0)

*v″*	0	*v″*	0
*v′*		*v′*	
0	9.0236–1	11	1.9235–11
1	9.0636–2	12	3.4295–12
2	6.5080–3	13	6.5247–13
3	4.5437–4	14	1.3180–13
4	3.5005–5	15	2.6924–14
5	3.1250–6	16	4.5920–15
6	3.2409–7	17	6.9115–16
7	3.8320–8	18	3.8920–16
8	5.0548–9	19	8.9534–16
9	7.3154–10	20	1.5161–15
10	1.1463–10	21	8.5939–16

**Table 9 t9-jresv65an5p451_a1b:** Franck-Condon Factors for the excitation of levels of N_2_^+^ (A^2^*II*) from N_2_(X^1^Σ_g_^+^, v=0)

*v*″	0
*v′*	
0	2.4468–1
1	3.1069–1
2	2.2530–1
3	1.2358–1
4	5.7340–2
5	2.3877–2

**Table 10:** Franck-Condon Factors for the excitation of levels of N_2_^+^(B^2^Σ_u_^+^) from N_2_(X^1^Σ_g_^+^, v=0)

*v″*	0	*v″*	0
*v′*		*v′*	
0	8.9119–1	15	1.7921–17
1	1.0703–1	16	2.8420–20
2	1.7514–3	17	2.9486–17
3	2.6855–5	18	1.0171–16
4	1.7966–6	19	1.3940–16
5	6.5482–8	20	2.0681–17
6	1.0485–9	21	3.5482–17
7	3.3727–10	22	1.7063–16
8	5.2112–12	23	2.6721–16
9	1.8534–13	24	1.3949–16
10	4.9245–14	25	5.5412–19
11	5.0633–17	26	9.7988–17
12	1.0188–15	27	3.1142–16
13	3.3279–16	28	4.3268–16
14	5.4866–17	29	2.8228–16
